# Diminished adrenal sensitivity to endogenous and exogenous adrenocorticotropic hormone in critical illness: a prospective cohort study

**DOI:** 10.1186/s13054-014-0721-8

**Published:** 2015-01-06

**Authors:** Margriet FC de Jong, Nienke Molenaar, Albertus Beishuizen, AB Johan Groeneveld

**Affiliations:** Department of Nephrology, VU University Medical Centre, De Boelelaan 1117, 1081HV Amsterdam, The Netherlands; Department of Surgery, University Medical Centre Groningen, Groningen, The Netherlands; Department of Intensive Care, Medical Spectrum Twente, Enschede, The Netherlands; Department of Intensive Care, VU University Medical Centre, Amsterdam, The Netherlands; Department of Intensive Care, Erasmus Medical Centre, Rotterdam, The Netherlands

## Abstract

**Introduction:**

Adrenal dysfunction may represent critical illness-related corticosteroid insufficiency (CIRCI), as evidenced by a diminished cortisol response to exogenous adrenocorticotropic hormone (ACTH), but this concept and its clinical significance remain highly controversial. We studied the adrenal response to exogenous ACTH as a function of the endogenous cortisol-to-ACTH ratio, a measure of adrenal sensitivity, and of clinical variables, during critical illness and recovery from the acute phase.

**Methods:**

We prospectively included 59 consecutive septic and nonseptic patients in the intensive care unit with treatment-insensitive hypotension in whom CIRCI was suspected; patients having received etomidate and prolonged corticosteroids were excluded. An ACTH test (250 μg) was performed, followed by a second test after ≥7 days in acute-phase survivors. Serum total and free cortisol, ACTH, and clinical variables were assessed. Patients were divided according to responses (delta, Δ) of cortisol to ACTH at the first and second tests.

**Results:**

Patients with low (<250 n*M*) Δ cortisol (*n* = 14 to 17) had higher baseline cortisol and ACTH but lower cortisol/ACTH ratios than patients with a normal Δ cortisol (≥250 n*M*) in the course of time. A low Δ cortisol in time was associated with more-severe disease, culture-positive sepsis, and prolonged activated prothrombin time. Results for free cortisol were similar.

**Conclusions:**

Even though the pituitary-adrenal axis is activated after stress during critical illness, diminished adrenal sensitivity to endogenous ACTH predicts a low increase of cortisol to exogenous ACTH, suggesting adrenal dysfunction, irrespective of the stage of disease. The data further suggest a role of disease severity and culture-positive sepsis.

## Introduction

The stress of critical illness, and sepsis in particular, strongly activates the hypothalamic-pituitary-adrenal (HPA) axis and increases circulating cortisol [1-9]. Nevertheless, the secretion and concentration of (free) cortisol may be relatively insufficient, even when not subnormal, for the severity of disease in the course of critical illness-related corticosteroid insufficiency (CIRCI), which may relate to morbidity and mortality, particularly in sepsis [2,3,6-15]. Albeit hotly debated, a relatively low increase in circulating cortisol on exogenous adrenocorticotropic hormone (ACTH) stimulation may denote adrenal dysfunction, which is associated with severe disease and ultimate mortality, and may warrant treatment with corticosteroids to increase survival from septic shock [2,6,7,11,12,14]. This may be accompanied by relatively high baseline cortisol levels but, as some studies suggest, by low cortisol/ACTH ratios, indicative of diminished adrenal sensitivity, even when ACTH levels do not exceed normal values [3,4,6,7,16-18]. Treatment with etomidate-inhibiting adrenal cortisol synthesis, indeed, reduces the cortisol/ACTH ratio and diminishes the cortisol response to exogenous ACTH [16,19]. The ratio is used to diagnose primary absolute adrenal insufficiency [20]. However, Boonen et al. [21] recently suggested that, during critical illness, impaired cortisol metabolism (measured in relatively few patients) contributes to elevated (free) cortisol levels and low endogenous ACTH levels via feedback inhibition, and thereby to a diminished response to exogenous ACTH in the absence of adrenal dysfunction. A diminished response to exogenous ACTH is, however, hard to explain on the basis of an acutely low endogenous ACTH level only. Nevertheless, decreased metabolism might explain in part the dissociation between low ACTH and elevated cortisol concentrations in persistent rather than acute (as in the Boonen et al. study [21]) critical illness over days, attributed to hypersensitivity or non-ACTH stimulation of the adrenals [6,22,23]. The controversy on adrenal function in critical illness is further compounded by the allegedly poor relation between protein-bound total and free cortisol involved in tissue effects and feedback, but increases in free cortisol may parallel increases in total cortisol, irrespective of binding proteins [5,17].

Finally, the question remains whether adrenal injury contributes to dysfunction, for instance, after diffuse intravascular coagulation and adrenal thrombosis or hemorrhage, as may occur during meningococcemia, other causes of sepsis, and heparin treatment, among others [2,16,24,25]. We previously showed in transversal but also in longitudinal, retrospectively analyzed observations that clinical predictors for a diminished cortisol response to exogenous ACTH are septic shock with severe disease, acidosis, and organ failure on the one hand and coagulation disturbances on the other [8,15]. In any case, a relatively low exogenous ACTH response during critical illness may normalize when disease severity resolves in surviving patients, whereas a decrease in response may be associated with increasing severity of disease and mortality, arguing against the ACTH test as a poorly reproducible laboratory phenomenon in the critically ill [15,26-28]. This may favor reversal and development of adrenal dysfunction in time, respectively.

The hypothesis for the present study is, first, that diminished adrenal sensitivity to endogenous ACTH attenuates responses to exogenous ACTH, irrespective of the stage of critical illness. Second, these abnormalities characterize adrenal dysfunction and insufficiency relative to disease severity in the course of critical illness. Third, sepsis and its sequelae is a main risk factor for adrenal dysfunction. We therefore examined these factors longitudinally in a cohort of critically ill patients who were able to undergo repeated ACTH testing.

## Materials and methods

### Patient population and ACTH test

**Figure 1:**
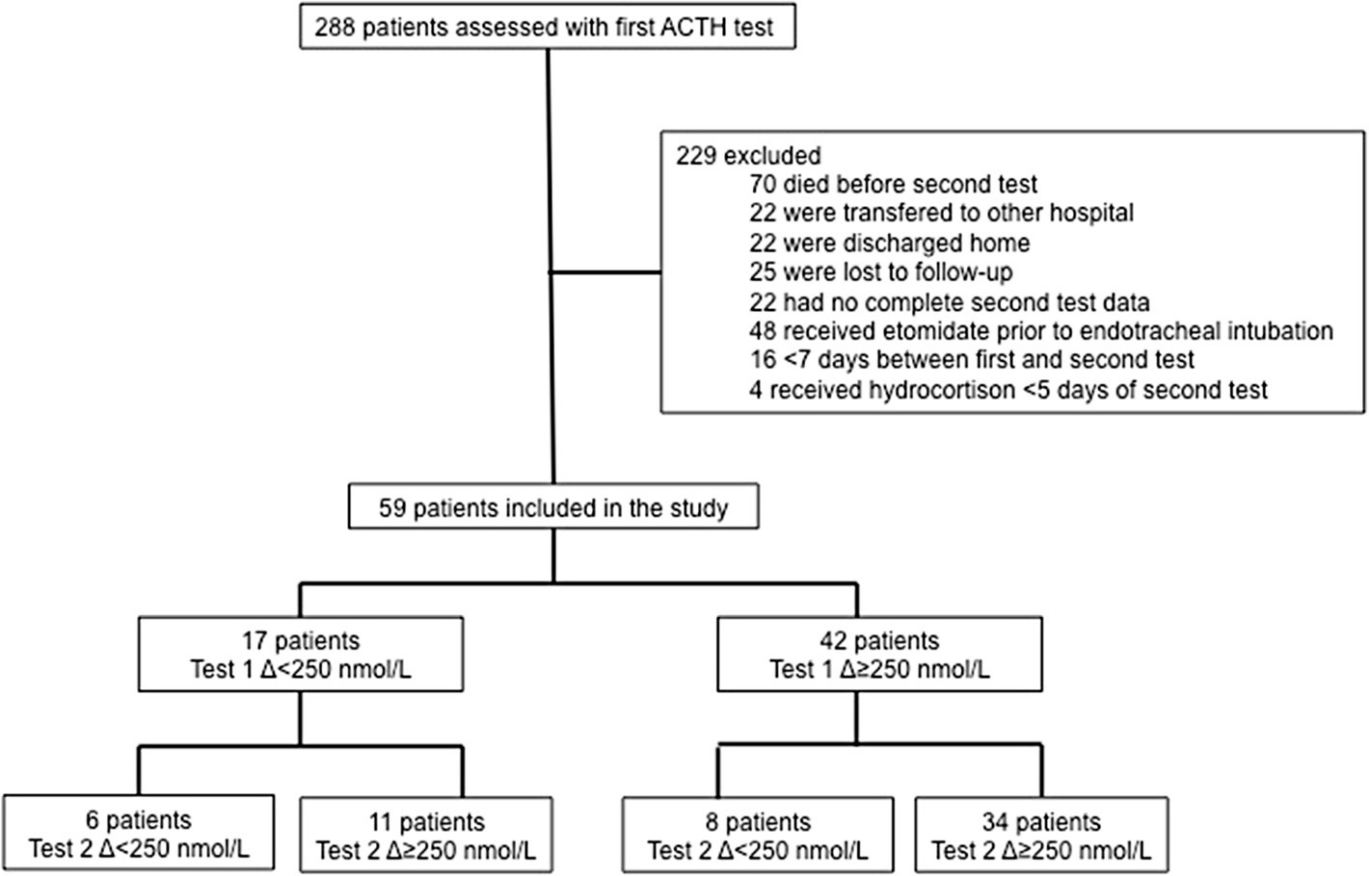
**Flow chart of patient inclusion for this study.**

This prospective study was carried out in the 32-bed general intensive care unit (ICU) of a university hospital from December 2004 to March 2007. According to Dutch legislation and the institutional ethical committee (Medical Ethical Committee VUmc, chair Prof dr JA Rauwerda), we did not need ethical approval and informed consent, because the ACTH test is routinely performed in our department for patient care, when clinically indicated, no extra blood was drawn for this study, and the results were treated anonymously. In the study period, an ACTH test was performed in 288 consecutive patients, and 59 patients (20%) were included when fulfilling criteria for the current study (Figure 1). These patients were older than 18 years, with a clinical suspicion of CIRCI on the basis of >6-hour hypotension (<100 mm Hg systolic) requiring repeated fluid challenges and/or vasopressor/inotropic treatment. They underwent a first ACTH test in the ICU (Test 1) and a second test (Test 2) before or after discharge from the ICU. A minimum interval of 7 days occurred between tests and a minimum interval of 5 days after cessation of hydrocortisone treatment when started. Patients were excluded if they had a history of HPA disease, if they received glucocorticoid treatment within 3 months of Test 1, or if they received etomidate before endotracheal intubation, because etomidate is a well-known inhibitor of adrenal function [2,13,14,16].

All patients underwent a short 250-μg ACTH (tetracosactide-hexa-acetate, Synacthen, Novartis Pharma, Basel, Switzerland) test. Blood samples were taken at baseline and 30 and 60 minutes after intravenous injection. Serum total cortisol was measured with competitive immunoassay (Advia Centaur; Siemens Diagnostics, Deerfield, IL, USA). The intra- and interassay coefficients of variation were 3% and 6%, respectively, and the detection limit was 30 n*M* (500 n*M* = 18 μg/dl). Serum free cortisol levels were measured with equilibrium dialysis of undiluted serum samples (*n* = 49 available) followed by radioimmunoassay (Orion Diagnostica, Espoo, Finland; <6% crossreactivity with cortisone). The intra- and interassay coefficients of variation were less than 7% and 8%, respectively. Samples for ACTH were immediately put on ice, and ACTH was determined with an immunometric assay and CBG with radioimmunoassay (Immulite 2500; Siemens Medical Diagnostic Solutions; *n* ACTH 2 to 26 p*M*; and BioSource, Nivelles, Belgium; *n* 30–54 mg/L, respectively). We calculated the ratio of baseline cortisol/ACTH as a parameter of adrenal sensitivity [3,4,16-18]. The peak (at 30 or 60 minutes) minus baseline cortisol level was taken to calculate increases on exogenous ACTH. A low response to ACTH in critical illness was defined by a subnormal total cortisol increase (<250 nmol/L) [6,7,10,12-14].

### Data collection

At study entry, the following parameters were recorded: time from ICU admission, age and sex, and ICD-10 diagnoses of common clinical conditions at admission. At the day of each ACTH test, the following were collected: serum total and free cortisol levels before and 30 and 60 minutes after 250 μg of i.v. ACTH, serum levels of ACTH cortisol-binding globulin (CBG), and albumin. Other laboratory measurements included total white blood cell count, platelet count, activated partial thromboplastin time (aPTT), and prothrombin time (PT). Interventions including type and doses of vasopressor/inotropes, intubation, need for mechanical ventilation, and renal replacement therapy were recorded. Sepsis was defined as the presence of systemic inflammatory response syndrome (SIRS) with a positive microbiologic local (urine, trachea, or other) and/or blood culture. SIRS was defined as two or more of the following criteria: a temperature of >38°C or <35.5°C, a leukocyte count >12 of <4 × 10^9^/L, a heart rate >90 per minute, and a respiratory rate >20 per minute, or the presence of mechanical ventilation. Suspected or microbiologically proven sources of sepsis were recorded. Disease severity was assessed by the acute physiology and chronic health evaluation score (APACHE II) and sequential organ-failure assessment score (SOFA) on the days of the ACTH test, and length of ICU stay and mortality in the ICU and hospital were recorded.

### Clinical management

Patients were treated according to local protocols, derived from national and international guidelines. Treatment of sepsis included antibiotics and drainage, whenever possible, and intubation, mechanical ventilation, and continuous veno-venous hemofiltration (CVVH) were instituted when needed. Patients and caregivers were not blinded to ACTH test results and moderate dose corticosteroids (hydrocortisone starting 100 mg TID i.v.) was administered at the discretion of attending intensivists. For anticoagulation, for instance during CVVH, mostly heparin was used.

### Statistical analysis

For categoric data the χ^2^ and Fisher Exact test were used (SPSS v21; IBM SPSS Inc, Chicago, IL, USA). Continuous variables were tested for normality with the Kolmogorov-Smirnov test, and if *P* < 0.05, they were logarithmically transformed for further analysis with the help of generalized estimating equations (GEEs). This was performed to investigate the association of Δ cortisol, time, and their first-order interaction to endocrine and clinical variables (in a linear model), taking repeated measurements in the same patients into account (in an exchangeable matrix). The Mann–Whitney *U* test was done for other group comparisons. The Spearman correlation coefficient (r_s_) was used to express the relation between pooled variables. Receiver operating characteristic area under the curve (AUROC) was used to evaluate predictive values. A two-sided *P* < 0.05 was considered to indicate statistical significance, and exact *P* values are given, unless <0.001. Data are expressed as median (range) or number (percentage).

## Results

### Patients

Patient characteristics are described in Table 1. In 14 (24%) patients, the second test was performed after discharge from the ICU. Six patients had in both tests Δ cortisol <250 n*M*, and 11 patients recovered and eight deteriorated in the course of time. Thirty-four patients had a normal response in both tests. All patients received hydrocortisone after the first test awaiting results.

**Table 1:** **Patient characteristics at admission**

	***n*** **= 59**
Age	68 (19–87)
Sex (m/f)	35 (59)/24(41)
Admission diagnosis	
Trauma and postoperative	23 (39)
Respiratory insufficiency	16 (27)
CPR	5 (9)
Sepsis	10 (17)
Renal insufficiency	4 (7)
Other	11 (19)

Median (range) or number (%) where appropriate. Patients may have more than two admission diagnoses. CPR, cardiopulmonary resuscitation.

### ACTH and cortisol

Levels of Δ cortisol as a continuous variable, which were directly associated with Δ free cortisol (*P* < 0.001), were inversely associated with ACTH levels irrespective of time (*P* < 0.001). Similarly, baseline cortisol and cortisol/ACTH were inversely and directly associated with Δ cortisol (*P* = 0.042 and 0.004), respectively. Test characteristics according to Δ cortisol <, ≥250 n*M* on test days are described in Table 2. It shows that the response in Δ (free) cortisol to exogenous ACTH was low when baseline ACTH as well as (free) cortisol levels were relatively high, but baseline (free) cortisol to ACTH ratios were low, as compared with normal responders to exogenous ACTH. CBG increased but ACTH and (free) cortisol did not decrease in time, in spite of decreasing indices of disease severity (Table 3).

**Table 2:** **ACTH Test 1 and 2 classified according to groups of Δ cortisol <, ≥ 250 n** ***M***

	**Test 1 Δ <250 n** ***M***	**Test 1 Δ ≥250 n** ***M***	**Test 2 Δ <250 n** ***M***	**Test 2 Δ ≥250 n** ***M***	***P*** **Δ cortisol, time, interaction**
	***n*** **= 17**	***n*** **= 42**	***n*** **= 14**	***n*** **= 45**	
ACTH, p*M*	3.1 (1.1-18.0)	1.5 (1.1-15.0)	6.0 (1.1-14.0)	3.8 (1.0-8.9)	0.001, 0.025, 0.72
Baseline cortisol, n*M*	470 (200–1,355)	430 (75–2,670)	655 (335–1,890)	495 (35–2,670)	0.021, 0.12, 0.30
Baseline cortisol/ACTH, n*M*/p*M*	148 (36–250)	238 (14–2,054)	109 (48–1,718)	126 (35–2,054)	0.029, 0.16, 0.14
Baseline free cortisol, n*M*	77 (63–357) (*n* = 9)	86 (38–256) (*n* = 14)	154 (114–252) (*n* = 6)	75 (4–151) (*n* = 20)	0.001, 0.91, 0.19
Baseline free cortisol/ACTH, n*M*/p*M*	22 (9–38)	39 (16–89)	26 (11–43)	17 (4–35)	0.042, 0.10, <0.001
Δ cortisol, n*M*	140 (−50-245)	388 (260–4,015)	177 (−80-230)	425 (255–965)	n.a., 0.45, 0.093
Δ free cortisol, n*M*	59 (−71-124)	151 (74–678)	40 (−34-99)	133 (32–233)	<0.001, 0.15, 0.16
CBG, mg/L	23 (6–43)	29 (13–63)	39 (4–60)	41 (21–65)	0.060, <0.001, 0.90
Albumin, g/L	13 (6–32)	17 (6–24)	16 (12–28)	20 (8–32)	0.25, <0.001, 0.89
Days from admission to test	1 (0–50)	3 (0–44)	25 (9–49)		
Days between tests	n.a.	n.a.	18 (8–34)	16 (8–43)	
Days after stopping hydrocortisone	n.a.	n.a.	7 (5–12)	7 (5–14)	0.75^a^

Median (range); ACTH, adrenocorticotropic hormone; CBG, cortisol-binding globulin. ^a^Mann-Whitney *U* test, otherwise generalized estimating equations; n.a., not applicable.

**Table 3:** **Clinical variables according to Δ cortisol <, ≥ 250 n** ***M***

	**Test 1 Δ < 250 n** ***M***	**Test 1 Δ ≥250 n** ***M***	**Test 2 Δ <250 n** ***M***	**Test 2 Δ ≥250 n** ***M***	***P*** **Δ cortisol, time, interaction**
	***n*** **= 17**	***n*** **= 42**	***n*** **= 14**	***n*** **= 45**	
**Disease severity**					
APACHE II	19 (14–33)	17 (5–31)	16 (10–24)	14 (5–21)	0.002, <0.001, 0.90
SOFA	9 (2–16)	9 (2–16)	5 (0–10)	4 (0–16)	0.46, <0.001, 0.91
MAP, mm Hg	71 (53–102)	80 (36–114)	83 (65–102)	87 (50–127)	0.20, 0.002, 0.61
Vasopressors/inotropes	13 (76)	35 (83)	4 (29)	9 (20)	0.85, <0.001, 0.56
MV	16 (94)	40 (95)	11 (79)	28 (62)	0.81, 0.002, 0.43
Renal replacement therapy	3 (17)	9 (20)	4 (29)	5 (11)	0.19, 0.88, 0.10
**Infection**					
Temperature, °C	36.8 (31.0-40.1)	36.9 (31.6-38.3)	37.1 (35.4-37.6)	36.8 (35.3-38.6)	1.0, 0.44, 0.83
Leukocytes, ×10^9^/L	13.4 (2.4-71.9)	13.3 (2.2-36.0)	12.7 (5.4-89.7)	9.9 (2.7-32.2)	0.35, 0.12, 0.82
SIRS	16 (94)	34 (81)	10 (71)	23 (51)	0.066, 0.002, 0.79
Sepsis	11 (65)	19 (45)	4 (29)	6 (13)	0.055, 0.001, 0.89
Positive cultures	11 (65)	20 (48)	4 (29)	8 (18)	0.15, 0.002, 0.88
**Coagulation**					
Platelets, ×10^9^/L	204 (59–661)	185 (51–803)	235 (24–419)	281 (88–721)	0.084, 0.19, 0.016
PT, INR	1.53 (0.99-2.47)	1.47 (1.05-4.77)	1.17 (0.98-5.46)	1.20 (1.02-3.04)	0.81, 0.068, 0.99
aPTT, seconds	60 (35–92)	48 (31–77)	55 (38–72)	51 (31–103)	0.14, 0.94, 0.037
**Outcome**					
Length of ICU stay, days	41 (11–130)	30 (3–104)	36 (13–92)	30 (3–130)	0.93^a^, 0.70^a^
Length of hospital stay, days	60 (21–230)	51 (15–141)	58 (31–230)	54 (15–175)	0.51^a^, 0.70^a^
ICU mortality	5 (29)	6 (14)	2 (14)	9 (20)	0.27b,1.00b
Hospital mortality	7 (41)	14 (33)	5 (36)	16 (36)	0.57b,1.00b

Median (range) or number (%), where appropriate; APACHE, acute physiology and chronic health evaluation; SOFA, sequential organ failure assessment; MAP: mean arterial pressure; MV, mechanical ventilation; SIRS, systemic inflammatory response syndrome; PT, prothrombin time; INR, international normalized ratio; aPTT: activated partial thromboplastin time; ICU, intensive care unit. ^a^Mann-Whitney *U* test, bFisher Exact test, otherwise generalized estimating equations.

### Clinical correlates

The following inversely related to Δ cortisol as a continuous variable (in GEE), independent of time: APACHE II (*P* < 0.001), renal replacement therapy (*P* = 0.047), sepsis (*P* = 0.055), positive cultures (0.041), and the aPTT (P = 0.020). In multiple GEE, a low Δ cortisol response was associated with sepsis (*P* = 0.003) and a low cortisol/ACTH ratio (*P* = 0.008) and their first-order interaction (*P* = 0.003). Clinical variables for Δ cortisol <, ≥250 n*M* on test days are described in Table 3. Low responders had elevated APACHE II scores and tended more often to have SIRS and sepsis, even though decreasing in time, than did normal responders. The aPTT was higher in the first test and the platelets in the second test in low versus normal responders (interaction between Δ cortisol and time). No differences in ICU or hospital mortality were found among response groups, nor in groups with persistently low or normal responses or changing from a low to normal response or vice versa.

### Correlations

**Figure 2:**
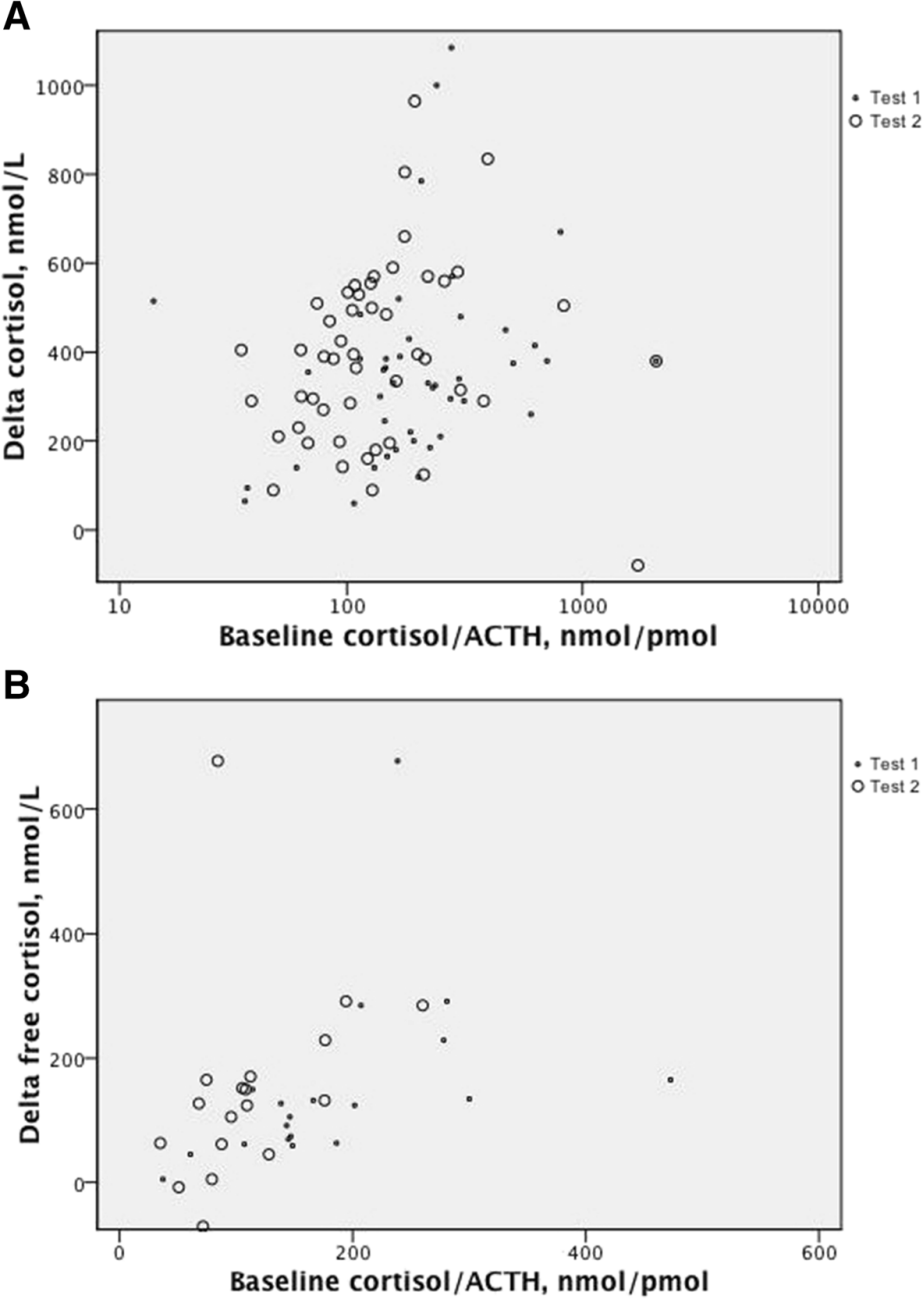
**The cortisol-to-ACTH ratio at baseline and the subsequent response in increase in circulating total (A) and free (B) cortisol with exogenous ACTH: r**_**s**_ **= 0.25 (r** ^**2**^ **= 0.06),** ***P*** **= 0.015; r**_**s**_ **= 0.53 (r** ^**2**^ **= 0.28),** ***P*** **< 0.001, respectively, together for Tests 1 and 2 in the course of critical illness (see text; no differences between tests).** This suggests that ACTH-insensitive adrenals respond less with cortisol secretion to exogenous ACTH.

Baseline total and free cortisol directly correlated at r_s_ = 0.78, *P* < 0.001; total and free Δ cortisol also correlated (r_s_ = 0.88; *P* < 0.001). At baseline, ACTH directly related to total and free cortisol (r_s_ = 0.34; *P* = 0.001 and r_s_ = 0.52; *P* = 0.001, respectively). Baseline ACTH inversely related to Δ total and free cortisol (r_s_ = −0.32, *P* = 0.002 and r_s_ = −0.49, *P* = 0.001, respectively). Baseline (free) cortisol inversely related to Δ (free) cortisol: min r_s_ = −0.26, *p* = 0.005 and max r_s_ = −0.45, *P* = 0.001. Baseline total cortisol/ACTH ratio directly related to Δ (free) cortisol (r_s_ = 0.25, *P* = 0.015 to r_s_ = 0.53, *P* < 0.001; Figure 2). Total and free cortisol/ACTH ratios directly correlated at r_s_ = 0.82, *P* < 0.001.

### Predictive values

Baseline ACTH and cortisol/ACTH predicted a low cortisol response to exogenous ACTH (AUROC, 0.72, *P* = 0.001, and 0.66, *P* = 0.017, respectively).

## Discussion

Our study suggests that, particularly in sepsis, diminished adrenal sensitivity to endogenous ACTH is associated with a low cortisol response to exogenous ACTH, irrespective of cortisol protein binding and stage of critical illness. This may denote adrenal dysfunction and insufficiency, relative to severity of disease.

Some studies suggest that baseline total cortisol is more elevated in patients subsequently responding less to exogenous ACTH with severe illness and elevated risk for death, than in those with normal baseline levels and responses [1,3,4,7,8,10,15,27]. In studies not reporting this relation, decreased binding molecules may have attenuated total cortisol levels [3,5-7,13,14]. Our study also suggests increasing baseline and decreasing Δ cortisol with increasing disease severity (APACHE II scores), so that adrenal function may not be well adapted to disease severity, thus suggesting insufficiency. Some studies evaluated baseline endogenous ACTH or cortisol/ACTH ratios and found relatively increased values and decreased ratios, respectively, at least in the acute phase of critical illness and predicting mortality [3,4,16-18]. Our ACTH levels are in the low-normal range, as reported before [6], but, nevertheless, predicted a low response to exogenous ACTH, irrespective of stage of disease. The latter, otherwise, renders unlikely a confounding effect of intercurrent hydrocortisone treatment. This is the first report, to the best of our knowledge, suggesting adrenal dysfunction on the basis of diminished adrenal sensitivity to endogenous and exogenous ACTH, as described after etomidate administration [16,19]. The time dependency of the association between a low free cortisol/ACTH ratio and a low cortisol response to exogenous ACTH can be attributed in part to the low number of observations available on free cortisol in low responders of Test 2. In any case, our results again suggest that (changes in) free cortisol parallel (changes in) total cortisol with ACTH testing, in spite of changing binding proteins in time [17]. Hence, free and total cortisol were almost interchangeable in the current evaluation.

Particularly during the chronic or prolonged phase of critical illness (rather than in the acute phase, as in the Boonen et al. study [20]), low ACTH and elevated cortisol levels can be seen, suggestive of increased sensitivity to ACTH or ACTH-independent cortisol production [2,21-23]. The latter may be caused by non-ACTH pituitary stimulants or feedback suppression of ACTH after impaired cortisol metabolism and persistent hypercortisolemia [2,21-23]. Our results do not suggest reversal or development of this so-called cortisol-ACTH dissociation, because the cortisol/ACTH ratio did not change in time. Boonen et al. [21] suggested that baseline cortisol was not under control of subnormal ACTH levels and that exogenous ACTH resulted in low responses of circulating cortisol, even though only peak and not Δs cortisol were reported. This was attributed, at least in part, to impaired cortisol metabolism with diminished feedback ACTH secretion.

However, lower endogenous ACTH levels in the course of panhypopituitarism or steroid treatment, for instance, variably diminish adrenal sensitivity to exogenous ACTH only after days to weeks [2,29]. In contrast, our correlation data suggest basal and particularly free cortisol levels being under persistent control of ACTH, as reported before [4,18]. Our results refute the impaired metabolism-low endogenous ACTH hypothesis in either the acute or the chronic phase of critical illness to explain fully a low Δ cortisol with exogenous ACTH, by suggesting that relatively low responses to exogenous ACTH are associated with diminished adrenal sensitivity to endogenous ACTH, irrespective of stage of disease. Nevertheless, the discrepancy between our study and the Boonen et al. study [19] is hard to explain, unless their ACTH values were diluted by more extensive fluid therapy or taken at different stages of disease than in our study.

Even though disease severity diminished in time in this selected cohort, the number of patients with subnormal ACTH responses only slightly decreased and tended to relate to persistent or new occurrence of SIRS and culture-positive sepsis and coagulation disturbances (prolonged aPTT), in line with other investigators suggesting sepsis as the main risk factor for adrenal dysfunction of the critically ill [3,6,7,9-14,18,26-28]. The currently prospectively studied clinical risk factors in time closely agree with those retrospectively found by us before, even in longitudinal analysis [8,15]. This favors the idea that a low Δ cortisol in time is of clinical significance, rather than reflecting poor reproducibility of the ACTH test.

Adrenal injury after adrenal thrombosis or hemorrhage may underlie the association of dysfunction with coagulation disturbances (for aPTT with Δ cortisol as a continuous variable), as described in meningococcemia and the Waterhouse-Friderichsen syndrome, because other infections may also cause adrenal thrombosis, hemorrhage, and insufficiency [2,16,24,25]. Heparin treatment also is a well-known risk factor [24].

The observed endocrine results did not bear any relation to mortality in the current study (as opposed to previous ones [1,3,7,8,10,12,13,15,27]), which may be explained by patient selection of acute-phase survivors allowing repeated ACTH testing within 3 weeks, on the average, by virtue of study design. Together with the exclusion of etomidate-treated patients, this selection may also explain the relatively low frequency of adrenal dysfunction of about 30% in our patients, whereas adrenal insufficiency may occur in up to 60% of those with septic shock [6-8,10-12,14,15,18,27]. Finally, we did not know or evaluate the potentially interfering phenomenon of steroid resistance in critical illness [30].

Our study has several limitations. First, we did not routinely measure cortisol metabolism or tissue effects in our patients because this requires elaborate techniques [8]. The patients studied are a selection because we wanted to evaluate repeated ACTH tests in various stages of disease.

## Conclusions

Our data in a large cohort of critically ill patients with follow-up ACTH testing suggest that, even though the HPA axis is activated during critical illness and recovery from the acute phase, diminished adrenal ACTH sensitivity predicts a low increase of cortisol to exogenous ACTH, suggesting adrenal dysfunction irrespective of stage of disease. The data further suggest a role of disease severity and culture-positive sepsis. The implications for treatment by corticosteroids remain unanswered by our study. Our study may nevertheless imply imaging the adrenals to look for hemorrhage or thrombosis in future studies on sepsis-induced adrenal dysfunction to help in deciding on corticosteroids.

## Key messages

- Even though the pituitary-adrenal axis is activated after stress in the course of critical illness, diminished adrenal sensitivity for endogenous ACTH is associated with a low increase of cortisol to exogenous ACTH.
- Diminished adrenal sensitivity to endogenous and exogenous ACTH suggests adrenal dysfunction.
- The data further suggest a role of disease severity and culture-positive sepsis in the adrenal dysfunction and insufficiency of the critically ill.

## Abbreviations

ACTH: Adrenocorticotropic hormone
APACHE II: Acute Physiology And Chronic Health Evaluation
aPTT: Activated partial thromboplastin time
AUROC: Receiver operating characteristic area under the curve
CBG: Cortisol-binding globulin
CIRCI: Critical illness-related corticosteroid insufficiency
CVVH: Continuous veno-venous hemofiltration
GEE: Generalized Estimating Equation
HPA: Hypothalamic-pituitary-adrenal
ICU: Intensive care unit
PT: Prothrombin time
r_s_: Spearman correlation coefficient
SOFA: Sequential organ-failure assessment
SIRS: Systemic inflammatory response syndrome
