# Erratum to: Diminished adrenal sensitivity to endogenous and exogenous adrenocorticotropic hormone in critical illness: a prospective cohort study

**DOI:** 10.1186/s13054-015-1015-5

**Published:** 2015-09-04

**Authors:** Margriet FC de Jong, Nienke Molenaar, Albertus Beishuizen, AB Johan Groeneveld

**Affiliations:** Department of Nephrology, VU University Medical Centre, De Boelelaan 1117, Amsterdam, 1081HV The Netherlands; Department of Surgery, University Medical Centre Groningen, Groningen, The Netherlands; Department of Intensive Care, Medical Spectrum Twente, Enschede, The Netherlands; Department of Intensive Care, VU University Medical Centre, Amsterdam, The Netherlands; Department of Intensive Care, Erasmus Medical Centre, Rotterdam, The Netherlands

Unfortunately, the original version of this article [1] contained an error. Table Two (Table 1 here) was included incorrectly. The correct Table Two (Table 1 here) can be found below:

**Table 1:** ACTH Test 1 and 2 classified according to groups of Δ cortisol <, ≥ 250 nmol/L

	Test 1 Δ<250 nmol/L	Test 1 Δ≥250 nmol/L	Test 2 Δ<250 nmol/L	Test 2 Δ≥250 nmol/L	P Δ cortisol, time, interaction
	n=17	n=42	n=14	n=45	
ACTH, pmol/L	3.1 (1.1–18.0)	1.5 (1.1–15.0)	6.0 (1.1–14.0)	3.8 (1.0–8.9)	0.001, 0.025, 0.72
Baseline cortisol, nmol/L	470 (200–1355)	430 (75–2670)	655 (335–1890)	495 (35–2670)	0.021, 0.12, 0.30
Baseline cortisol/ACTH, nmol/pmol	148 (36–250)	238 (14–2054)	109 (48–1718)	126 (35–2054)	0.029, 0.16, 0.14
Baseline free cortisol, nmol/L	77 (63–357) (n=9)	86 (38–256) (n=14)	154 (114–252) (n=6)	75 (4–151) (n=20)	0.001, 0.91, 0.19
Baseline free cortisol/ACTH, nmol/pmol	22 (9–38)	39 (16–89)	26 (11–43)	17 (4–35)	0.042, 0.10, <0.001
Δ cortisol, nmol/L	140 (−50–245)	388 (260–4015)	177 (−80–230)	425 (255–965)	n.a., 0.45, 0.093
Δ free cortisol, nmol/L	59 (−71–124)	151 (74–678)	40 (−34–99)	133 (32–233)	<0.001, 0.15, 0.16
CBG, mg/L	23 (6–43)	29 (13–63)	39 (4–60)	41 (21–65)	0.060, <0.001, 0.90
Albumin, g/L	13 (6–32)	17 (6–24)	16 (12–28)	20 (8–32)	0.25, <0.001, 0.89
Days from admission - test	1 (0–50)	3 (0–44)	25 (9–49)	20 (9–83)	0.57*, 0.15*
Days between tests	n.a.	n.a.	18 (8–34)	16 (8–43)	0.41*
Days after stop hydrocortisone	n.a.	n.a.	7 (5–12)	7 (5–14)	0.75*

Median (range); ACTH: adrenocorticotropic hormone, CBG: cortisol binding globulin. *Mann–Whitney U test, otherwise generalized estimating equations; n.a.: not applicable
